# Association between gallstones and subclinical coronary atherosclerosis: An observational study with propensity score matching

**DOI:** 10.1371/journal.pone.0356142

**Published:** 2026-08-14

**Authors:** Soyoung Kim, Hoonsub So, Hyun Woo Park, Sangwoo Park, Seung Bum Lee, Soe Hee Ann, Sung Wook Hwang, Young-Jee Jeon, Eun Ji Park, Woon Jung Kwon, Seong Hoon Choi, Seok Won Jung, Sung-Jo Bang, Gyung-Min Park

**Affiliations:** 1 Department of Internal Medicine, Ulsan University Hospital, University of Ulsan College of Medicine, Ulsan, Republic of Korea; 2 Department of Internal Medicine, Soonchunhyang University Bucheon Hospital, Soonchunhyang University College of Medicine, Bucheon, Republic of Korea; 3 Department of Internal Medicine, Asan Medical Center, University of Ulsan College of Medicine, Seoul, Republic of Korea; 4 Department of Family Medicine, Ulsan University Hospital, University of Ulsan College of Medicine, Ulsan, Republic of Korea; 5 BigData Center, Ulsan University Hospital, University of Ulsan College of Medicine, Ulsan, Republic of Korea; 6 Department of Diagnostic Radiology, Ulsan University Hospital, University of Ulsan College of Medicine, Ulsan, Republic of Korea; 7 Basic-Clinic Convergence Research Institute, University of Ulsan, Ulsan, Republic of Korea; Charite Universitatsmedizin Berlin, GERMANY

## Abstract

Previous studies reporting an association between gallstones and coronary artery disease have not considered the types of coronary artery plaque. Additionally, the association between gallstones and subclinical coronary atherosclerosis has not been clearly elucidated. Therefore, this study aimed to analyze the association between gallstones and subclinical coronary atherosclerosis based on the type of coronary artery plaque in asymptomatic Koreans using coronary computed tomography angiography. We retrospectively analyzed 9,113 individuals (mean age, 53.7 ± 8.0 years; 5,907 men [64.8%]) who had no history of coronary artery disease and underwent a general medical checkup. Gallstones were diagnosed by transabdominal ultrasonography. Coronary computed tomographic angiography was used to evaluate the type of coronary artery plaque and the severity of subclinical coronary atherosclerosis, and a diameter stenosis of ≥50% was considered clinically significant. Logistic regression and propensity score matching analyses were performed to determine the association between gallstones and subclinical coronary atherosclerosis. Among the study participants, 503 (5.5%) had gallstones. After adjusting for risk factors, significant associations were found between gallstones and noncalcified plaque (adjusted odds ratio [OR], 1.728; 95% confidence interval [CI] 1.267–2.357; p = 0.001), as well as between gallstones and significant coronary artery stenosis (adjusted OR, 1.386; 95% CI, 1.002–1.917; p = 0.049). In asymptomatic individuals, gallstones were independently associated with noncalcified plaque (the high-risk plaque) and significant coronary artery stenosis which is often linked to poorer cardiac outcomes. It is crucial to acknowledge the potential for subclinical coronary atherosclerosis and to effectively manage cardiovascular risk factors in individuals with gallstones.

## Introduction

Coronary artery disease (CAD) stands as a leading global cause of mortality [1]. There is a high demand for effective and cost-efficient preventions and treatments to decrease the risk of CAD [2]. Age, male, obesity, smoking, diabetes mellitus, hypertension, and hyperlipidemia are risk factors for CAD [3–5]. Controlling its risk factors, such as weight control, smoking cessation, treatment of diabetes mellitus, hypertension, and hyperlipidemia is key to the prevention and management of CAD [6–10]. However, the exact understanding of CAD risk factors remains incomplete.

Gallstones have been suggested to be associated with CAD. Risk factors for gallstone disease include age, female, rapid weight loss, overweight or obese, and certain medical conditions, such as diabetes mellitus and liver disease [11]. Although gallstones and CAD share common risk factors, several studies have reported an association between gallstones and CAD even after adjustment for these confounding factors [12,13]. However, in a large cohort of Korean adults, Kwon et al. found no significant association between gallstone disease and coronary artery calcification (CAC), which is a marker of subclinical atherosclerosis and a significant predictor of future CAD [14]. We hypothesized that the inconsistency was caused by the types of coronary artery plaque. Previous studies reporting an association between gallstones and CAD have not considered the types of coronary artery plaque. Additionally, the association between gallstones and subclinical coronary atherosclerosis has not been clearly elucidated. Therefore, this study aimed to analyze the association between gallstones and subclinical coronary atherosclerosis in asymptomatic Koreans using coronary computed tomography angiography (CCTA).

## Methods

### Ethics statements

This retrospective study was approved by the local Institutional Review Board of Ulsan University Hospital, Ulsan, Korea (IRB file No. UUH 2023-07-065). The requirement for patients’ informed consent was waived due to the retrospective design of this study. The data underlying the findings of this study are maintained by the Ulsan University Hospital Institutional Data Access Committee and made available only to researchers who meet the criteria for access to confidential data. Having met these institutional access criteria, the authors accessed the coded data for research purposes from August 24, 2023, to February 28, 2024.

### Study population

**Fig 1:**
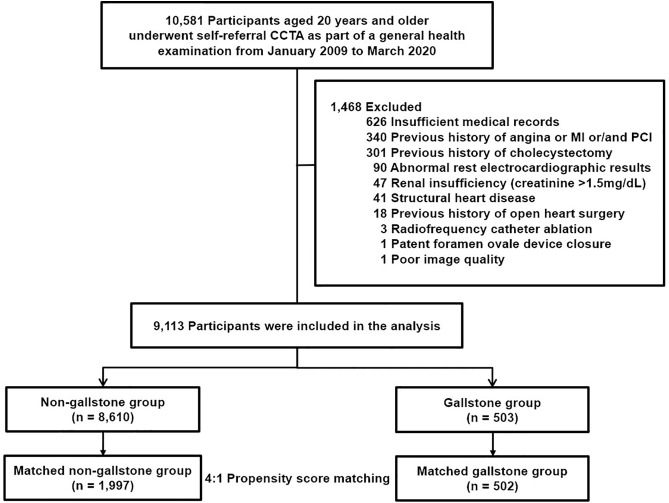
Flow chart of the participant selection process. CCTA, coronary computed tomographic angiography; MI, myocardial infarction; PCI, percutaneous coronary intervention.

A retrospective analysis was conducted on 10,581 Korean individuals aged ≥20 years who underwent self-referral CCTA as a part of general health examination at Ulsan University Hospital’s Health Promotion Center between January 2009 and March 2020. Fig 1 presents the participant selection process in detail. A total of 9,113 individuals were included in this study after excluding ineligible individuals.

### Clinical and laboratory measurements

The clinical and laboratory data were obtained from the participants’ electronic medical records and Ulsan University Hospital’s clinical data warehouse platform [15,16]. During the general health examination, body weight, height, waist circumference, and blood pressure were measured using standard methods as previously described [15,16]. Blood samples taken after an overnight fast were analyzed for levels of hemoglobin A1c, fasting glucose, total cholesterol, triglycerides, low-density lipoprotein cholesterol, high-density lipoprotein cholesterol (HDL-C), creatinine, uric acid, bilirubin, aspartate aminotransferase, alanine aminotransferase, gamma-glutamyl transpeptidase, alkaline phosphatase, and C-reactive protein (CRP). Every participant underwent standard 12-lead electrocardiography. Echocardiography was also performed to evaluate the left ventricular ejection fraction.

According to the World Health Organization’s recommended Asian-specific cutoff value, obesity was classified as a body mass index of ≥25 kg/m^2^ [15,16]. Diabetes mellitus was defined as having a hemoglobin A1c level of ≥6.5%, a fasting plasma glucose level of ≥126 mg/dL, or a self-reported history of diabetes mellitus and/or treatment of diabetes mellitus with antidiabetic medication or dietary modification [15,16]. Hypertension was defined as having a self-reported history of hypertension with or without the use of antihypertensive medication, systolic blood pressure of ≥140 mmHg, or diastolic blood pressure of ≥90 mmHg [15,16]. Hyperlipidemia was defined as either a self-reported history of hyperlipidemia and/or use of antihyperlipidemic treatment or a total cholesterol level of ≥240 mg/dL [15,16]. If individuals had a first-degree relative with CAD, they were considered to have a family history of CAD [17]. The 10-year CAD risk for each participant was computed on the basis of previous studies’ calculations [3,4].

### Evaluation of gallstones

Gallstones were identified using ultrasonography after overnight fasting. The ultrasonographic evaluation was performed using an iU22 ultrasound scanner or an Affiniti 70G ultrasound scanner with a C5-1 convex transducer (Philips Healthcare, Bothell, WA, USA). The ultrasonographic characteristics of gallstones include the presence of posterior acoustic shadowing, gallstone mobility with changes in the individual’s position, and a highly reflective echo from the anterior surface of the gallstone [18].

### CCTA image acquisition and analysis

CCTA was performed using either a single-source 256-slice computed tomography (CT) scanner (Brilliance iCT; Philips Healthcare, Best, Netherlands) or a dual-source CT equipment (Somatom Definition Flash; Siemens, Erlangen, Germany). As previously described [15,16], a standard scanning protocol was applied. Interpretation of all CCTA images and calcium scoring were carried out by a cardiologist and experienced cardiovascular radiologists (G.M.P., W.J.K., and S.H.C., each with >10 years of experience). This analysis was performed using a dedicated workstation (syngo.via; Siemens or Aquarius iNtuition; TeraRecon, Durham, NC, USA) and followed the guideline provided by the Society of Cardiovascular Computed Tomography [19]. The coronary artery calcium score (CACS) was determined in accordance with previously described methods, and participants categorized based on their CACS: 0, 1–10, 11–100, 101–400, and >400 [20,21]. Types of coronary artery plaque were defined as follows: 1) calcified plaque: plaques with calcified tissue representing >50% of the plaque area (density >130 Hounsﬁeld units); 2) mixed plaque: plaque with <50% calcium; 3) noncalcified plaque: plaque without calcium [22]. A diameter stenosis of ≥50% was defined as clinically significant [22].

### Statistical analysis

Continuous variables were compared using the unpaired Student’s t-test or nonparametric Mann–Whitney U test, and categorical variables were compared using the chi-square test or Fisher’s exact test, as appropriate. We selected age, sex, obesity, diabetes mellitus, hypertension, hyperlipidemia, current smoker, family history of CAD, and CRP level as clinically significant variables based on previous epidemiologic studies [5,15,16]. Multivariable logistic regression analysis was performed using these covariates. A propensity score matching analysis was conducted on the basis of the variables listed in Table 1 to minimize the influence of potential confounding factors between the gallstone group and the non-gallstone group. We matched the individuals with gallstones versus those without gallstones using the one-to-four nearest-neighbor propensity score matching method within a caliper width equal to 0.2. Covariate balance was assessed by comparing the standardized mean differences between the groups after matching. The standardized mean differences in the baseline variables were <0.1 (10%) (S1 Fig). The sufficient overlap of propensity score distributions between the gallstone group and the non-gallstone group was verified after matching (S2 Fig). Logistic regression analysis with generalized estimating equations for categorical variables that considered the clustering of the matched pairs was used to analyze the risk of subclinical coronary atherosclerosis within the propensity score-matched population. To ensure model stability in the logistic regression analyses, we evaluated the events-per-variable (EPV) ratio, with a threshold of 10 or higher indicating sufficient stability. The data was managed and statistical analyses were conducted using R software (version 4.0.2; R Foundation for Statistical Computing, Vienna, Austria; www.r-project.org) and SPSS software (version 24; IBM Corp., Armonk, NY, USA). The R “MatchIt” package was used to conduct the propensity score matching. All statistical analyses were two-sided, and a *p* value less than 0.05 was considered to indicate a statistically significant relationship.

**Table 1:** Baseline characteristics of the study population according to the presence of gallstones.

Characteristic	Overall population (n = 9,113)				Propensity score-matched population (n = 2,499)			
	Overall (n = 9,113)	Non-gallstone group (n = 8,610)	Gallstone group (n = 503)	*p* value	Overall (n = 2,499)	Non-gallstone group (n = 1,997)	Gallstone group (n = 502)	*p* value
Age, years	53.7 ± 8.0	53.6 ± 8.0	56.0 ± 8.0	<0.001	55.8 ± 7.9	55.7 ± 7.9	56.0 ± 8.0	0.572
Male sex, n (%)	5,907 (64.8)	5,581 (64.8)	326 (64.8)	0.997	1,614 (64.6)	1,289 (64.5)	325 (64.7)	0.922
Body mass index, kg/m^2^	24.2 ± 2.9	24.2 ± 2.9	24.6 ± 3.0	<0.001	24.6 ± 3.0	24.6 ± 2.9	24.6 ± 3.0	0.919
Waist circumference, cm	85.6 ± 7.8	85.5 ± 7.8	87.3 ± 7.9	<0.001	87.3 ± 7.7	87.3 ± 7.7	87.3 ± 7.9	0.976
Systolic blood pressure, mmHg	124.7 ± 13.8	124.5 ± 13.8	127.9 ± 13.8	<0.001	127.7 ± 13.8	127.7 ± 13.8	127.9 ± 13.9	0.769
Diastolic blood pressure, mmHg	78.4 ± 9.4	78.4 ± 9.4	79.4 ± 9.3	0.016	79.4 ± 9.2	79.4 ± 9.2	79.4 ± 9.3	0.972
Current smoker, n (%)	1,989 (22.2)	1,869 (22.1)	120 (24.3)	0.246	609 (24.4)	490 (24.5)	119 (23.7)	0.729
Obesity, n (%)	3,329 (36.5)	3,110 (36.1)	219 (43.5)	0.001	1,086 (43.5)	867 (43.4)	219 (43.6)	0.624
Diabetes mellitus, n (%)	1,162 (13.0)	1,066 (12.6)	96 (19.4)	<0.001	489 (19.6)	394 (19.7)	95 (18.9)	0.686
Hypertension, n (%)	3,012 (33.5)	2,799 (33.0)	213 (42.8)	<0.001	1,053 (42.1)	837 (41.9)	216 (43.0)	0.614
Hyperlipidemia, n (%)	1,620 (18.1)	1,532 (18.1)	88 (17.7)	0.826	454 (18.2)	366 (18.3)	88 (17.5)	0.667
Family history of CAD, n (%)	804 (9.0)	759 (9.0)	45 (9.1)	0.960	242 (9.7)	196 (9.8)	46 (9.2)	0.653
Hemoglobin A1c level, %	5.7 ± 0.9	5.7 ± 0.8	5.8 ± 0.9	<0.001	5.8 ± 0.9	5.8 ± 0.9	5.8 ± 0.9	0.677
Fasting blood glucose level, mg/dL	96.3 ± 22.4	96.1 ± 21.9	100.7 ± 30.1	<0.001	101.0 ± 27.7	101.1 ± 27.1	100.5 ± 29.9	0.686
Total cholesterol level, mg/dL	191.6 ± 36.7	191.9 ± 36.6	187.9 ± 37.4	0.018	187.7 ± 36.7	187.7 ± 36.5	187.7 ± 37.3	0.994
Triglyceride level, mg/dL	116.5 ± 77.5	116.3 ± 78.0	119.7 ± 68.1	0.330	122.0 ± 75.0	122.5 ± 76.6	119.7 ± 68.2	0.410
LDL cholesterol level, mg/dL	127.5 ± 34.1	127.6 ± 34.0	125.5 ± 34.7	0.175	125.3 ± 33.8	125.3 ± 33.6	125.6 ± 34.7	0.850
HDL cholesterol level, mg/dL	52.8 ± 14.8	52.9 ± 14.8	50.7 ± 15.17	<0.001	50.2 ± 13.8	50.2 ± 13.7	50.4 ± 14.4	0.694
Creatinine level, mg/dL	0.85 ± 0.18	0.85 ± 0.18	0.85 ± 0.20	0.691	0.85 ± 0.19	0.85 ± 0.19	0.85 ± 0.20	0.735
Uric acid level, mg/dL	5.36 ± 1.36	5.37 ± 1.36	5.30 ± 1.33	0.247	5.28 ± 1.34	5.27 ± 1.34	5.30 ± 1.33	0.914
Bilirubin level, mg/dL	0.89 ± 0.39	0.89 ± 0.39	0.90 ± 0.41	0.561	0.90 ± 0.43	0.90 ± 0.43	0.90 ± 0.41	0.938
Aspartate aminotransferase level, IU/L	25.0- ± 13.2	25.0 ± 13.3	25.3 ± 12.1	0.630	25.2 ± 11.7	25.2 ± 11.6	25.2 ± 12.0	0.943
Alanine aminotransferase level, IU/L	27.2 ± 19.2	27.1 ± 18.9	28.5 ± 23.7	0.117	28.5 ± 19.9	28.5 ± 18.9	28.3 ± 23.5	0.874
Gamma-glutamyl transpeptidase level, IU/L	39.2 ± 52.4	38.8 ± 45.9	45.1 ± 117.3	0.551	40.7 ± 44.8	40.8 ± 44.3	40.3 ± 46.8	0.831
Alkaline phosphatase level, IU/L	64.0 ± 18.8	63.8 ± 18.6	66.8 ± 22.0	0.005	66.2 ± 19.1	66.2 ± 19.0	66.3 ± 19.3	0.950
C-reactive protein level ≥2 mg/L	48 (0.5)	46 (0.5)	2 (0.4)	0.999	15 (0.6)	13 (0.7)	2 (0.4)	0.517
Ejection fraction, %	64.4 ± 4.6	64.3 ± 4.6	64.6 ± 4.1	0.393	64.6 ± 4.3	64.6 ± 4.2	64.6 ± 4.4	0.930
Framingham risk score	6.9 ± 5.0	6.8 ± 4.9	8.6 ± 5.8	<0.001	8.6 ± 5.6	8.6 ± 5.6	8.6 ± 5.8	0.920
ASCVD risk score	6.6 ± 6.7	6.5 ± 6.6	8.8 ± 8.0	<0.001	8.6 ± 8.0	8.6 ± 8.1	8.8 ± 7.9	0.594

Values are shown as mean ± standard deviation or number (%).

CAD, coronary artery disease; HDL, high-density lipoprotein; LDL, low-density lipoprotein; ASCVD, atherosclerotic cardiovascular disease.

## Results

### Baseline characteristics

The study population had a mean age of 53.7 ± 8.0 years, with 5,907 (64.8%) male participants. Among the study participants, 503 (5.5%) had gallstones. Table 1 presents the baseline characteristics of the participants according to the presence or absence of gallstones. Individuals with gallstones were older and had higher body mass index, waist circumference, systolic blood pressure, and diastolic blood pressure than those without gallstones. The prevalences of obesity, diabetes mellitus, and hypertension were higher in the gallstone group than in the non-gallstone group. Higher levels of hemoglobin A1c, fasting blood glucose, and alkaline phosphatase were also observed in the study participants with gallstones than in those without. In contrast, lower total cholesterol and HDL-C levels were observed in individuals with gallstones than in those without. After 4:1 propensity score matching, there were 2,499 matched participants. The matched study population had a mean age of 55.8 ± 7.9 years, with 1,614 (64.6%) male participants. There were no significant differences in the covariates between the participants with and without gallstones in the matched pairs (Table 1). The number of events for any atherosclerotic plaque, calcified plaque, noncalcified plaque, mixed plaque, and significant coronary artery stenosis in propensity score-matched population is presented in S1 Table.

### CCTA findings

Table 2 presents the CCTA findings of the study population based on the presence or absence of gallstones. The study population’s mean CACS was 37.8 ± 153.1. Individuals with gallstones had a higher CACS than those without (*p* < 0.001). Coronary plaques were detected in 3,044 (33.4%) individuals. Specifically, 2,810 (30.8%), 540 (5.9%), and 335 (3.7%) individuals had calcified, noncalcified, and mixed plaques, respectively. The gallstone group had a higher prevalence of any coronary, calciﬁed, noncalciﬁed, or mixed plaque compared with the non-gallstone group (all, *p* < 0.05). Additionally, 558 individuals (6.1%) had at least one signiﬁcant coronary artery stenosis, and the prevalence of significant coronary artery stenosis was also higher in individuals with gallstones than in those without (*p* < 0.001).

**Table 2:** Coronary computed tomographic angiographic findings according to the presence of gallstones.

Variable	Overall population (n = 9,113)			
	Overall (n = 9,113)	Non-gallstone group (n = 8,610)	Gallstone group (n = 503)	*p* value
Mean coronary artery calcium score	37.8 ± 153.1	36.5 ± 149.6	59.8 ± 202.8	<0.001
Coronary artery calcium score classification, n (%)				<0.001
0	6,238 (68.5)	5,941 (69.0)	297 (59.0)	
1–10	782 (8.6)	740 (8.6)	42 (8.3)	
11–100	1,282 (14.1)	1,193 (13.9)	89 (17.7)	
101–400	596 (6.5)	540 (6.3)	56 (11.1)	
> 400	210 (2.3)	191 (2.2)	19 (3.8)	
Any coronary plaque, n (%)	3,044 (33.4)	2,829 (32.9)	215 (42.7)	<0.001
Plaque characteristic, n (%)				
Calcified plaque	2,810 (30.8)	2,608 (30.3)	202 (40.2)	<0.001
Noncalcified plaque	540 (5.9)	486 (5.6)	54 (10.7)	<0.001
Mixed plaque	335 (3.7)	304 (3.5)	31 (6.2)	0.002
Significant stenosis, n (%)	558 (6.1)	507 (5.9)	51 (10.1)	<0.001

Values are shown as mean ± standard deviation or n (%).

### Association between gallstones and subclinical coronary atherosclerosis

In univariable analyses, individuals with gallstones had significantly higher odds of any coronary plaque, calcified plaque, noncalcified plaque, mixed plaque, and significant coronary artery stenosis compared with those without (all, *p* < 0.05; Table 3). After adjusting for risk factors, including age, male, obesity, diabetes mellitus, hypertension, hyperlipidemia, current smoker, family history of CAD, and CRP level, no statistically significant differences were observed in the odds of any coronary plaque (adjusted odds ratio [OR], 1.190; 95% confidence interval [CI], 0.966–1.466; *p* = 0.103), calcified plaque (adjusted OR, 1.211; 95% CI, 0.982–1.493; *p* = 0.074), and mixed plaque (adjusted OR, 1.272; 95% CI, 0.841–1.924; *p* = 0.255) between the two groups. However, the odds of noncalcified plaque (adjusted OR, 1.728; 95% CI, 1.267–2.357; *p* = 0.001) and significant coronary artery stenosis (adjusted OR, 1.386; 95% CI, 1.002–1.917; *p* = 0.049) were significantly higher in the gallstone group than in the non-gallstone group (Table 3). In the propensity score-matched population (n = 2,499), there was a signiﬁcant association between gallstones and noncalciﬁed plaque (OR, 1.563; 95% CI, 1.125–2.172; *p* = 0.008), as well as between gallstones and significant coronary artery stenosis (OR, 1.401; 95% CI, 1.013–1.939; *p* = 0.042) (Table 3).

**Table 3:** Association between gallstone and coronary computed tomography angiographic findings.

Variable	Univariable analysis		Multivariable analysis		Propensity score matching analysis	
	Odds ratio (95% CI)	*p* value	Adjusted odds ratio (95% CI)	*p* value	Odds ratio (95% CI)	*p* value
Any atherosclerotic plaque						
Gallstone group	1.526 (1.271–1.831)	<0.001	1.190 (0.966–1.466)	0.103	1.149 (0.945–1.398)	0.164
Non-gallstone group	1	–	1	–	1	–
Calcified plaque						
Gallstone group	1.544 (1.285–1.857)	<0.001	1.211 (0.982–1.493)	0.074	1.186 (0.972–1.447)	0.092
Non-gallstone group	1	–	1	–	1	–
Noncalcified plaque						
Gallstone group	2.010 (1.494–2.705)	<0.001	1.728 (1.267–2.357)	0.001	1.563 (1.125–2.172)	0.008
Non-gallstone group	1	–	1	–	1	–
Mixed plaque						
Gallstone group	1.794 (1.226–2.627)	0.003	1.272 (0.841–1.924)	0.255	1.499 (0.991–2.267)	0.055
Non-gallstone group	1	–	1	–	1	–
Significant stenosis						
Gallstone group	1.803 (1.332–2.442)	<0.001	1.386 (1.002–1.917)	0.049	1.401 (1.013–1.939)	0.042
Non-gallstone group	1	–	1	–	1	–

CI, confidence interval.

Covariates in the multivariable model included age, sex, obesity, diabetes mellitus, hypertension, hyperlipidemia, current smoker, family history of coronary artery disease, and C-reactive protein level ≥2 mg/L

## Discussion

This study showed an association between gallstones and subclinical coronary atherosclerosis, particularly noncalcified plaques and significant coronary artery stenosis, after adjusting for cardiovascular risk factors.

Some studies have reported the association between gallstones and subclinical atherosclerosis. A cross-sectional study conducted by Méndez-Sánchez et al. showed the association of gallstones with carotid atherosclerosis, which was evaluated by carotid artery intima-media thickness [23]. Another study by Serin et al. found an association between gallstones and abdominopelvic artery atherosclerosis, assessed using abdominal ultrasonography [24]. Additionally, Yu et al. suggested that gallstone disease predicted arterial stiffness progression measured by brachial-ankle pulse wave velocity (baPWV) [25]. Carotid artery intima-media thickness, abdominopelvic artery atherosclerosis by ultrasonography, the ankle-brachial index test, and baPWV are indirect measures used to assess the atherosclerotic burden in the coronary artery [26,27]. However, few studies have investigated the association between gallstones and direct markers of coronary atherosclerosis. Even in the study by Kwon et al., no significant association was observed between gallstones and CAC, which is one of the direct markers of coronary atherosclerosis [14]. Notably, gallstones were not significantly associated with CAC, whereas they were associated with other subclinical atherosclerosis. We hypothesized that the inconsistency was caused by the types of coronary artery plaque. To our knowledge, no previous studies have evaluated the association between gallstones and the types of coronary artery plaque. Therefore, this study analyzed the association between gallstones and subclinical coronary atherosclerosis according to the types of coronary artery plaque.

Generally, noncalcified plaques are considered vulnerable and have a stronger association with acute coronary syndrome than calcified plaques [28,29]. A nationwide population-based sample cohort study, using the Korean National Health Insurance Service, reported that patients with gallstones had a higher risk of acute coronary syndrome than those without gallstones (hazard ratio (HR), 1.30; 95% CI, 1.15–1.47; *p* < 0.0001) [30]. Our study showed that gallstones are associated with noncalcified plaques, which may suggest a potential mechanism underlying the association between gallstones and acute coronary syndrome. Therefore, individuals with gallstones should pay more attention to the possibility of CAD.

Several studies have reported an association between gallstones and an increased long-term risk of CAD. Zheng et al. followed up with approximately 270,000 adults for ≤30 years and found that participants with a history of gallstones had a 23% higher risk of developing coronary heart disease than those without gallstones [13]. Another large prospective cohort study conducted by Lv et al. also reported an increased risk of ischemic heart disease in individuals with gallstones (HR, 1.23; 95% CI, 1.17–1.28), even after adjusting for established risk factors [31]. A meta-analysis conducted by Fan et al., which included eight cohort studies of nearly one million participants, showed that individuals with a medical history of gallstone disease had a significantly increased risk of fatal and nonfatal cardiovascular events than individuals without gallstone disease [12]. Our study identified an association between gallstones and significant coronary artery stenosis. Subclinical atherosclerosis can lead to the occurrence of cardiovascular events, causing substantial cardiovascular morbidity and mortality [32]. Early detection and appropriate preventive interventions for subclinical atherosclerosis are crucial to prevent future cardiovascular events [33,34]. Since gallstones could be associated with significant coronary artery stenosis, individuals with gallstones should be monitored closely for early detection of subclinical coronary atherosclerosis. Additionally, they should be vigilant in managing their cardiovascular risk factors. Nevertheless, the association between gallstones and significant coronary artery stenosis should be interpreted with caution. The multivariable analysis yielded a p-value close to the statistical threshold (p = 0.049). The risk of a Type I error cannot be entirely ruled out. Therefore, this particular finding should be considered exploratory and necessitates validation in future prospective trials.

The mechanism underlying the association between gallstones and atherosclerosis is not yet fully understood. However, there are a few possible explanations. Farnesoid X receptor (FXR), the bile acid-activated nuclear receptor, plays a critical role in regulating bile acid homeostasis [35,36]. Impaired FXR signaling leads to decreased bile acid secretion and altered phospholipid composition, which accelerates gallstone formation [37]. Interestingly, prior animal models have demonstrated that the activation of FXR significantly reduces the development of atherosclerosis by regulating plasma lipoprotein clearance and suppressing vascular inflammation [38]. Therefore, gallstones and atherosclerosis may share a common underlying pathophysiology related to FXR-related signaling, altered bile acid signaling, and lipid metabolism. Furthermore, transmembrane G-coupled protein receptor 5 (TGR5) is another significant component of the bile acid signaling network. [39] The research using TGR5 loss- and gain-of-function mouse models showed that TGR5 activation inhibits the production of inflammatory cytokines, suppresses oxidized LDL uptake in macrophages, and alleviates cellular stress, thereby attenuating atherosclerosis. [39–41] These mechanisms could link gallstones to atherosclerosis, especially noncalcified plaque that is lipid-rich. However, it should be noted that these plausible mechanisms have not been directly examined in the present study.

This study had some limitations. First, because our study design was cross-sectional, a cause-and-effect relationship between gallstones and subclinical coronary atherosclerosis could not be established. Additionally, because this study was conducted on asymptomatic individuals who underwent CCTA, we were unable to elucidate the association between gallstones and cardiovascular events. Second, although we adjusted for clinically important variables, there may have been unmeasured confounding factors. Nevertheless, we tried to mitigate this limitation by using propensity score matching analysis. Third, only information about the presence of gallstones was available; information about stone number, size, type, symptoms, duration, and prior biliary events was unavailable or imprecise. This limited phenotyping may have introduced exposure heterogeneity or misclassification, as different stone types or disease durations may have varying degrees of metabolic and inflammatory impacts on atherosclerosis. Fourth, there is a possibility of selection bias as the study population voluntarily participated in health checkups. Further multicenter prospective studies are necessary to analyze the association between gallstones and cardiovascular events, considering the types of gallstones and coronary artery plaque.

In conclusion, there is a significant association between gallstones and subclinical coronary atherosclerosis in the Korean population, specifically caused by noncalcified plaques and significant coronary artery stenosis. Therefore, it is crucial to be aware of the possibility of subclinical coronary atherosclerosis and properly manage cardiovascular risk factors in individuals with gallstones.

## Supporting information

S1 Fig Covariate balance before and after propensity score matching. (TIF)

S2 Fig Propensity score distributions and overlap before and after matching. (TIF)

S1 Table The number of events in propensity score-matched population. (TIF)
